# Total thyroidectomy: reduction in postoperative hypoparathyroidism

**DOI:** 10.1530/EC-23-0198

**Published:** 2023-08-24

**Authors:** Rasmus Reinke, Stefano Christian Londero, Martin Almquist, Lars Rejnmark, Lars Rolighed

**Affiliations:** 1 Department of Otorhinolaryngology, Head and Neck Surgery, Aarhus University Hospital, Aarhus, Denmark; 2 Department of Surgery, Lund University Hospital, Lund, Sweden; 3 Department of Endocrinology and Internal Medicine, Aarhus University Hospital, Aarhus, Denmark

**Keywords:** hypoparathyroidism, thyroid cancer, surgery, total thyroidectomy, parathyroid, Graves’ disease, goitre

## Abstract

**Objective:**

Total thyroidectomy is associated with a high risk of postoperative hypoparathyroidism, mainly due to the unintended surgical damage to the parathyroid glands or their blood supply. It is possible that surgeons who also perform parathyroid surgery see lower rates of postoperative hypoparathyroidism. In a single institution, we investigated the effects of restricting total thyroidectomy operations for Graves’ disease to two surgeons who performed both thyroid and parathyroid surgeries. We aimed to evaluate the rates of postoperative hypoparathyroidism in a 10-year period with primary attention toward patients with Graves’ disease.

**Design:**

Retrospective cohort study from a single institution.

**Methods:**

We defined the rate of permanent hypoparathyroidism after total thyroidectomy as the need for active vitamin D 6 months postoperatively. Between 2012 and 2016, seven surgeons performed all thyroidectomies. From January 2017, only surgeons also performing parathyroid surgery carried out thyroidectomies for Graves’ disease.

**Results:**

We performed total thyroidectomy in 543 patients. The rate of permanent hypoparathyroidism decreased from 28% in 2012–2014 to 6% in 2020–2021. For patients with Graves’ disease, the rate of permanent hypoparathyroidism decreased from 36% (13 out of 36) in 2015–2016 to 2% (1 out of 56) in 2020–2021. In cancer patients, the rate of permanent hypoparathyroidism decreased from 30% (14 out of 46) in 2012–2014 to 10% (10 out of 51) in 2020–2021.

**Conclusion:**

Restricting thyroidectomy to surgeons who also performed parathyroid operations reduced postoperative hypoparathyroidism markedly. Accordingly, we recommend centralisation of the most difficult thyroid operations to centres and surgeons with extensive experience in parathyroid surgery.

**Significance statement:**

Thyroid surgery is performed by many different surgeons with marked differences in outcome. Indeed, the risk of postoperative permanent hypoparathyroidism may be very high in low-volume centres. This serious condition affects the quality of life and increases long-term morbidity and the patients develop a life-long dependency of medical treatments. We encountered a high risk of hypoparathyroidism after the operation for Graves’ disease and restricted the number of surgeons to two for these operations. Further, these surgeons were experienced in both thyroid and parathyroid surgeries. We show a dramatic reduction in postoperative hypoparathyroidism after this change. Accordingly, we recommend centralisation of total thyroidectomy to surgeons with experience in both thyroid and parathyroid procedures.

## Introduction

Total thyroidectomy (TT) is associated with a high risk of permanent postoperative hypoPT (hypoPT) (1, 2). TT is the most common cause of hypoPT due to unintentional resection or surgical damage of the parathyroid glands (PG) or their vascular supply (3). HypoPT may reduce quality of life (4) and increases both mortality (5) and morbidity (6). Morbidity for patients with hypoPT includes an increased risk of developing chronic kidney disease (CKD) and kidney calcifications (7). Finally, hypoPT results in a longer hospital stay (8) after TT.

The frequency of postoperative hypocalcaemia and/or hypoPT after TT varies significantly in the literature. This variation is thought to be at least partly due to differences in the definitions used (9). In recent years, many studies defined postoperative hypoPT as the need for active vitamin D and/or calcium for more than 6 or 12 months postoperatively (10, 11, 12, 13).

At Aarhus University Hospital, Denmark, TT was performed by surgeons who did and who did not also perform parathyroid surgery. Our own data showed a higher rate of permanent hypoPT after TT, especially for Graves’ disease (GD), when performed by surgeons who did not also perform parathyroid surgery. Hence, in January 2017, it was decided that only surgeons who also perform parathyroid surgery were allowed to carry out total thyroidectomy for Graves’ disease. The aim of the present study was to compare the rate of both temporary and permanent hypoPT between different time periods, between different indications for thyroid surgery and between different surgeons.

## Materials and methods

### Cohort

This is a retrospective cohort study from Aarhus University Hospital, Department of Otorhinolaryngology, Denmark. We identified all patients who underwent TT (procedure code KBAA60) between January 1, 2012, and December 31, 2021. Patients were excluded if the thyroidectomy was performed in more than one step or if the operation was part of an additional procedure that would compromise parathyroid function, e.g. laryngectomy or if parathyroid glands were intentionally removed for the treatment of hyperparathyroidism. We included patients with TT who also underwent central and/or lateral lymph node resections. Finally, we evaluated biochemistry and postoperative ordinations in all patients with at least 6 months of follow-up.

### Groups and time periods

We classified thyroidectomies according to the aetiology (goitre/GD/cancer) based on the indication to undergo thyroid surgery. We also subdivided operations according to the type of surgeon (surgeons who did or did not also perform parathyroid surgery). Furthermore, we divided operations into four time periods according to the changes in the allocation of surgeons and changes in the clinical management of the patients. In the first period (2012–2014), the annual volume of TT in the institution was below 30 and surgeons had only thyroid surgical experience. In the second period (2015–2016), the annual volume increased to around 65 TTs due to referrals from general surgical departments. In the third period (2017–2019), attention was attracted toward hypoPT, and surgeons with parathyroid experience (>100 parathyroid procedures per year and >100 thyroid procedures per year) performed an increasing proportion of TT procedures and the majority of TT for patients with GD. In the last period (2020–2021), the majority of TT procedures were performed by surgeons who also did parathyroidectomies (80%). Both groups had large experience in thyroid surgery, and the volume of thyroidectomies was in favour of the thyroid-only surgeons in the first two periods.

### Patients and treatments

All data from the first two time periods (2012–2016) were analysed retrospectively. From January 2017, all thyroid operations were entered prospectively in a local quality registry. Since January 2017, all patients have had biochemical measurements once or twice daily including ionised calcium (Ca^2+^) and parathyroid hormone (PTH). Furthermore, we coordinated the follow-up of all patients in the Department of Endocrinology in the case of postoperative hypocalcaemia. Treatment with alfacalcidol was initiated on postoperative days one to three in the case of Ca^2+^ values below 1.10 mmol/L.

### Definitions

We defined normocalcaemia as plasma levels of Ca^2+^ in the reference range (1.18–1.32 mmol/L). Accordingly, hypocalcaemia was defined as Ca^2+^ below the reference range (<1.18 mmol/L). We defined temporary hypoPT as postoperative hypocalcaemia after TT, but with normalisation within 6 months and the ability to withdraw medical treatment. Permanent hypoPT was defined as postoperative hypocalcaemia where the level of PTH was insufficient to normalise Ca^2+^ within 6 months. These patients had ongoing alfacalcidol treatment, and there had been unsuccessful attempts to reduce this treatment. Despite normalisation of PTH in the follow-up period, the hypoPT diagnosis was maintained if patients continued on alfacalcidol supplementation. The current definition is supported by recent recommendations (14).

No ethics approval was obtained for this study, as The Central Denmark Region Committees on Health Research Ethics did not classify the study as a health research study According to the Consolidation Act on Research Ethics Review of Health Research Projects, Consolidation Act number 1338 of 1 September 2020 section 14. The case number for this study is 1-10-72-1-21 in the ethics committee.

## Results

### Whole dataset

**Figure 1:**
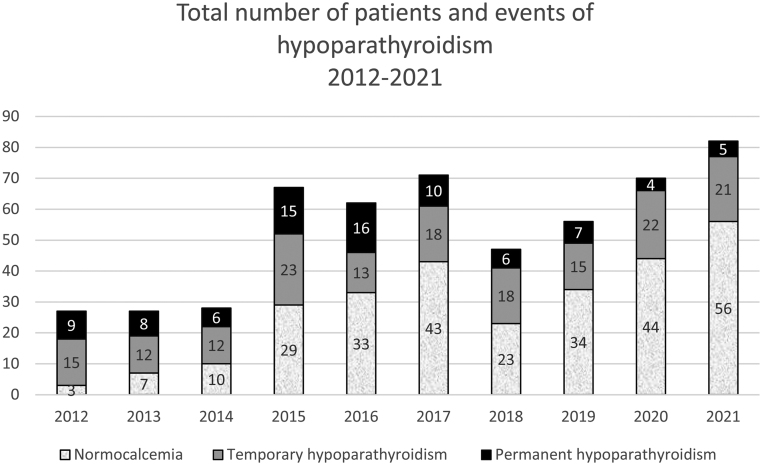
Number of patients with total thyroidectomies in Aarhus University Hospital 2012–2021 and hypoparathyroidism status after 6 months.

**Figure 2:**
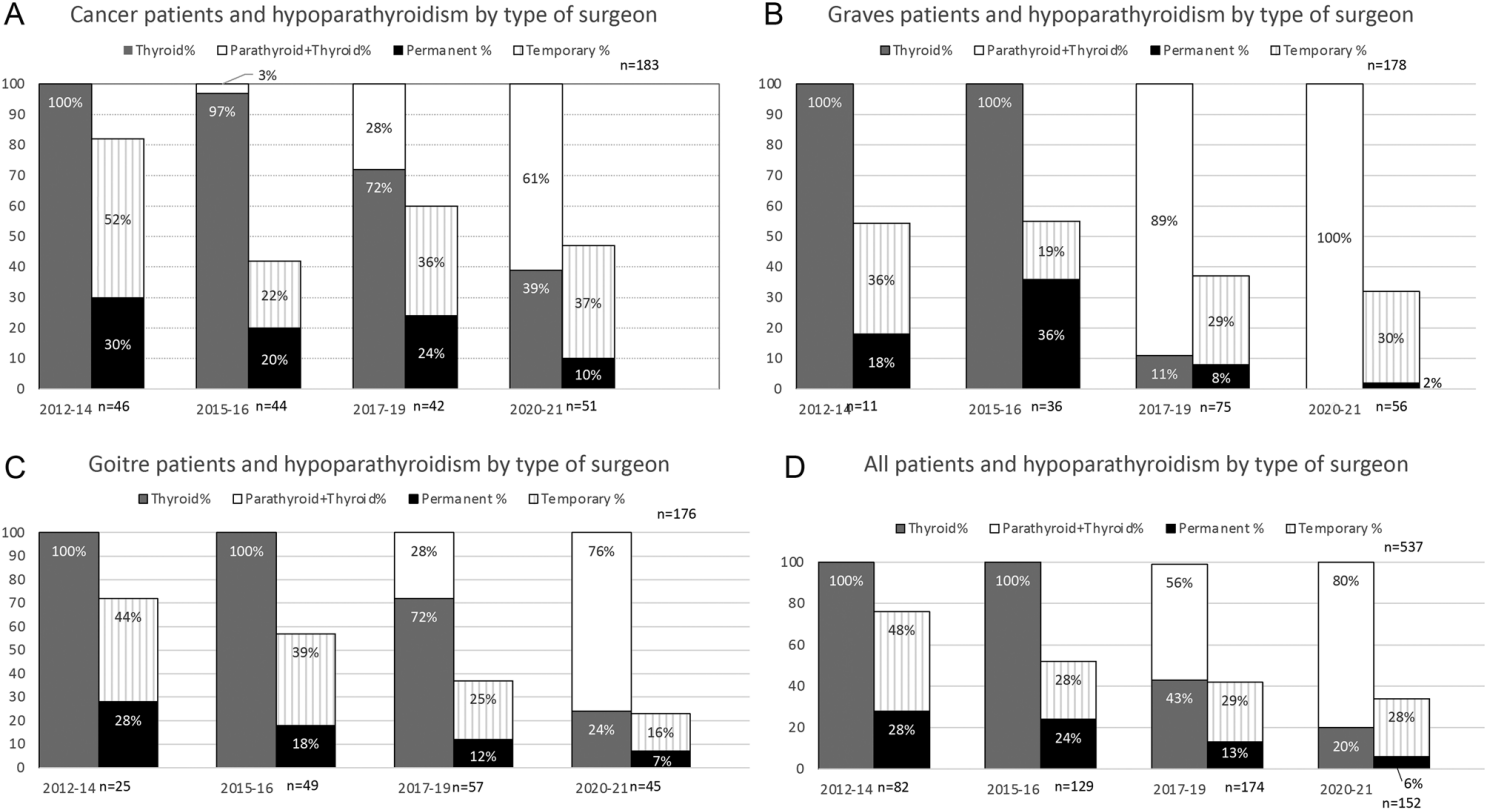
Total thyroidectomies performed by either thyroid surgeons or parathyroid + thyroid surgeons in (A) cancer patients, (B) Graves’ patients and (C) goitre patients over time. (D) Distribution of surgeon over time and hypoparathyroidism from 2012 to 2021.

A total of 543 patients underwent TT during the study period. A total of six patients were excluded, two had their TT procedure performed in multiple steps and another two had concomitant laryngectomies. One had hypercalcaemia of malignancy before and after TT. The last excluded patient also underwent intended total parathyroidectomy due to CKD. For included patients (*N* = 537), indications for surgery were either goitre (*n* = 176), GD (*n* = 178) or cancer (*n* = 183). A total of 85 patients (16%) developed permanent hypoPT and 169 patients (31%) had temporary hypoPT (Fig. 1). The risk of permanent hypoPT for all patients (Table 1) was highest (21%) when the indication for surgery was cancer. The risk of temporary hypoPT for this group was 37%. For goitre, the risk of temporary and permanent hypoPT was 29% and 15%, respectively. For GD, the risk of temporary and permanent hypoPT was 28% and 12%, respectively. When allocating the patients according to the type of surgeon, 59% were operated on by a thyroid surgeon and 41% by a surgeon who performed both parathyroid and thyroid surgeries. The risk of temporary and permanent hypoPT for the thyroid surgeon was 34% and 23%, respectively. The risk of temporary and permanent hypoPT for the surgeon who performed both parathyroid and thyroid surgeries was 28% and 5%, respectively (Table 2, Fig. 2D).

**Table 1:** Indications for surgery and outcomes.

Label	Normocalcaemia	Temporary	Permanent	Total
Aetiology				
Goitre	99 (56%)	51 (29%)	26 (15%)	176
Graves’ disease	106 (60%)	50 (28%)	22 (12%)	178
Cancer	77 (42%)	68 (37%)	38 (21%)	183
Total	282	169	86	537

**Table 2:** Type of surgeon and outcomes.

Label	Normocalcaemia	Temporary	Permanent	Total
Type of surgeon				
Thyroid	137 (43%)	107 (34%)	74 (23%)	318
Parathyroid + thyroid	145 (66%)	62 (28%)	12 (5%)	219
Total	282	169	86	537

**Figure 3:**
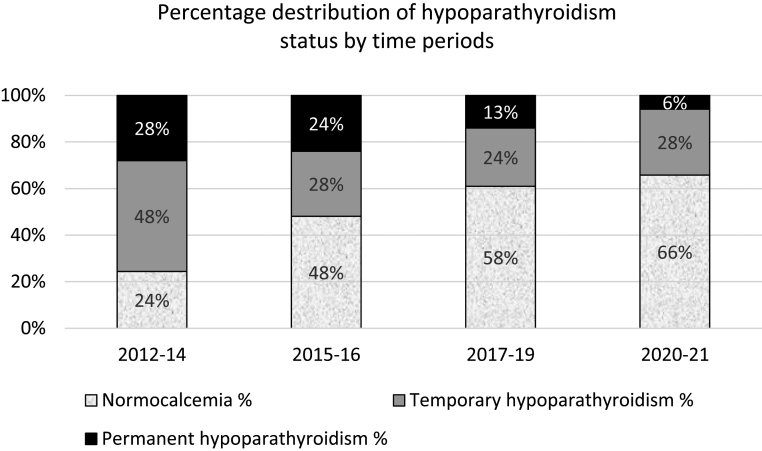
Percentage-wise distribution of calcium status 6 months post-surgery after total thyroidectomy in Aarhus University Hospital 2012–2021.

Over time, the risk of hypoPT decreased. When looking at all patients in the selected time periods, the risk of temporary and permanent hypoPT decreased, respectively, from 47% and 28% in 2012–2014 to 28% and 6% in 2020–2021, respectively. In the same periods, TT was performed more frequently (0% to 83%) by surgeons who also perform parathyroid surgery (Fig. 3). The relative risk reduction for the development of permanent postoperative hypoPT from the first 5 years (2012–2016) to the last 5 years (2017–2021) was for all patients 62%. The same relative risk reduction for patients with GD in the same period was 83%.

### Graves’ disease

For patients with GD, the risk of hypoPT over time can be seen in Fig. 2B. In 2012–2014 the risk of temporary and permanent hypoPT was 36% and 18%, respectively. In 2015–2016 ,19% of patients had temporary hypoPT and 36% permanent hypoPT. With a change in surgeons in January 2017, the risk of temporary and permanent hypoPT decreased to 29% and 8% in 2017–2019 and 30% and 2% in 2020–2021, respectively. From 2017 to 2021, the percentage of GD patients operated by parathyroid surgeons increased from 79% in 2017–2019 to 100% in 2020–2021. In that same time period, the risk of permanent hypoPT decreased markedly and only 1 out of 56 patients operated on in the last 2 years still needs alfacalcidol.

### Cancer patients

For patients with cancer, the risk of hypoPT decreased over time (Fig. 2A). From 2012–2014 to 2020–2021 the frequency of temporary and permanent hypoPT was reduced from 52% and 30% to 37% and 10%, respectively. From 2012 to 2016, no patients undergoing TT for cancer were operated by a surgeon with experience in parathyroidectomy. From 2017 to 2021, the percentage of parathyroid surgeons operating cancer patients increased from 12% in 2017 to 68.0% in 2021. In that same time period, the risk of permanent hypoPT reduced from 35% in 2017 to 8% in 2021.

### Goitre patients

The risk of temporary and permanent hypoPT in goitre patients decreased from 44.0% and 28.0% in 2012–2014 to 16% and 7% in 2020–2021, respectively (Fig. 2C). Similarly, the frequency of postoperative normocalcaemia after TT increased from 28% to 78%. No patients undergoing TT for goitre were operated on by a surgeon who also operates on parathyroids in 2012–2016 and the risk of permanent hypoPT in this period went from 31% in 2012 to 27% in 2016. From 2017 to 2021, the percentage of goitre patients operated by surgeons who also operate on parathyroids increased from 19% in 2017 to 79% in 2021.

## Discussion

In the last 10 years, we found the risk of permanent hypoPT after TT decreased markedly. Depending on various indications for TT, permanent hypoPT was reduced from more than 30% to around 5% in the study period. The intervention for this improvement was changing thyroid-only surgeons to surgeons experienced in thyroid and parathyroid surgeries, but several other factors may be involved.

Regardless of the reasons for this improvement, the majority of the patients today undergo surgery with no impact on calcium levels, require only a short postoperative hospital stay and do not suffer from the long-term morbidity associated with hypoPT.

Increased morbidity and mortality have been linked to postoperative hypoPT (5, 6, 15, 16, 17, 18), this fact could also have contributed to increased surgical awareness to preserve the PG. The morbidity is primarily associated with reduced quality of life and reduced kidney function (4, 7, 16, 19). Other forms of long-term morbidity from hypoPT include ectopic calcifications (17). These calcifications may arise due to the treatment of hypoPT with high doses of calcium and vitamin D. Furthermore, with low levels of PTH, the phosphate levels may increase and cause an increased calcium–phosphate product which over time leads to calcifications. Interestingly, attempts have been made to evaluate whether pre-operative treatment with calcium and vitamin D can affect the postoperative frequency of hypoPT. A recent systematic review from 2021 Khatiwada and Harris showed that in seven out of nine trials, with pre-operative supplementation with hypercalcaemic drugs (calcium, vitamin D and derivatives or calcium-sparing diuretics), the rate of postoperative hypocalcaemia was reduced (20). Conversely, Kanan *et al.* and Erlem *et al.* showed no significant effects of optimisation of 25-hydroxyvitamin D levels by pre-operative vitamin D supplementation on postoperative hypocalcaemia (21, 22).

In patients with GD, Hungry Bone Syndrome may occasionally contribute to a postoperative decrease in calcium levels. Especially in this patient group, parathyroid preservation can be more surgically challenging as the thyroid gland has increased vascularity. Edafe *et al.* showed that preoperative treatment of vitamin D deficiency in patients with GD reduces the risks of postoperative hypocalcaemia and hypoPT (23). However, despite improved vitamin D levels, the main goal during the operation is still to identify and preserve the parathyroid glands. We showed that limiting in the number of surgeons also allowed for a higher volume of operations for each surgeon. This was also described in the recommendations and guidelines (24). Furthermore, we showed that for surgeons with extensive experience in parathyroidectomy, the quality of TT improved due to a markedly reduced frequency of hypoPT. The main reason for this improvement may simply be increased attention toward the parathyroid glands and greater experience in identifying and preserving these glands. So far, we have not seen similar studies on differences in outcomes based on the differences in surgical training.

New studies on the parathyroid glands have disclosed a previously unknown autofluorescent ability of the gland (25). This parathyroid autofluorescence can be seen with a specialised camera using near-infrared fluorescence imaging (NIR) where the parathyroid glands can be distinguished clearly from surrounding tissues (25). In a prospective study, the postoperative risk of hypocalcaemia was reduced markedly after NIR cameras with parathyroid autofluorescence were introduced (26). This result seems similar to our current results when comparing between thyroid and parathyroid surgeons. Furthermore, the first result from a randomised controlled trial with NIR was published just a few years ago (27). The use of fluorescence was also introduced in this centre in November 2020. We cannot exclude that the use of fluorescence in a small sample of cases could have contributed to the reduced risk of postoperative hypoPT in the last year. However, the vast majority of patient operations did not involve the use of fluorescence.

Attention was also attracted toward the postoperative treatment with alfacalcidol and the need for a gradual reduction in the daily dose postoperatively. It is well known that the majority of patients with postoperative hypocalcaemia only have temporary hypoPT. However, if Ca^2+^ and PTH levels return to normal in the postoperative months, then treatment with alfacalcidol must be reduced accordingly. This improvement may take several months. In these postoperative months, the number of patients with a need for alfacalcidol slowly decreases. If the definition of hypoPT is based on the need for alfacalcidol after 6 months, the hypoPT percentage will be higher than after 12 months. These differences in the duration of alfacalcidol treatment differ in different guidelines (14, 28). We defined hypoPT as the need for alfacalcidol more than 6 months after TT. However, some of the patients in the current hypoPT group were on low alfacalcidol doses and had PTH levels in the normal range, which would have been classified differently according to other definitions. This was also seen in a similar Dutch study showing a hypoPT rate of 28% after 6 months and 15% after 1 year (10).

Our study has some limitations and the results reported must be interpreted while considering the known weaknesses of a retrospective cohort study. Second, being a single centre makes the sample size small. The patients included in this study were found by procedure codes in hospital journals; therefore, missing or erroneous codes would lead to exclusion from the study. However, these procedure codes are used to determine the diagnose-related groups taxation rate which funds the public healthcare system in Denmark and is considered to be accurate. Furthermore, patients were excluded if the thyroidectomy was performed in more than one step. Recent studies have shown that patients undergoing completion thyroidectomies seem to have a lower risk of postoperative hypoPT than patients having a total thyroidectomy in one step (29, 30). Therefore, we consider the risks of these procedures to be incomparable.

A strength of the study is the systematic read of all patient files, medical treatment and biochemistry with follow-up of each patient post-surgery and after at least 6 months to determine if the patient was still being prescribed active vitamin D and was thereby still classified as having hypoPT. Further, the medical treatments were ordinated by specialised endocrinologists without awareness of the current study.

## Conclusion

Restricting thyroidectomy to surgeons with extensive experience in parathyroid surgery drastically reduced postoperative hypoparathyroidism. Accordingly, we recommend centralising the most difficult thyroid surgeries to centres and surgeons with extensive experience in parathyroid surgery.

## Declaration of interest

The authors declare that there is no conflict of interest.

## Funding

Funding for this study was received from the fund for the advancement of health research in Central Denmark Region.

## Author contribution statement

L Rolighed designed the study and R Reinke drafted the final manuscript. M Almqust, L Rejnmark and S Londero advised on design and participated in writing the manuscript.
